# Blood-Letting

**Published:** 1887-08-20

**Authors:** 


### The Doctor's Pulpit.

### BLOOD-LETTING.

It is a circumstance worthy of some attention that doctors, who as a class are Conservative in politics, should have so great a contempt for the traditions of their own profession. The poorest village apothecary of these latter days will pour scorn on the treatment of the greatest lights of the medicine of a hundred years ago. Is the parish Æsculapius really a great philosopher, and was his by no means remote predecessor entirely an unredeemed blockhead? We do not for a moment believe it. In an address on medicine delivered before the meeting of the British Medical Association at Dublin, Professor W. T. Gairdner gave some extracts from the lectures of the famous Gregory, which probably made the hair of some of his less educated hearers stand on end, as much with excessive self-admiration as with horror at the barbarity and dangerousness of the treatment recorded. Gregory was a famous physician, and, like Cullen, a notorious bleeder. "By far the most effectual way of checking inflammation of the lungs," says Gregory, "is blood-letting. In most cases more than one bleeding is required, though some very mild cases may be treated by one bleeding, and that not a very large one. I have seen some patients cured by a single blood-letting, to the extent of ten or twelve ounces only; but it is very seldom that practitioners are called in early enough to effect a cure so easily. . . . *Six, eight, or ten times in the course of two or three weeks* is commonly sufficient to stop an ordinary pneumonia. It is often found necessary to bleed twice, sometimes three or four times in one day. But this very rough practice is by no means to be employed without a very sufficient reason. . . . We must always be aware of the necessity of not urging it too far."

There stands the judgment of one of the most capable and experienced men of his day, and that day not more than sixty or seventy years ago. Was the man an absolutely ignorant fool, teaching other fools how to kill their fellow-men most promptly *secundum artem?* The question is absurd! For our part we would rather have placed ourselves in the hands of Gregory, blood-letting and all, than in the hands of any merely routine practitioner of the present or any other day. What we assert is, that an honest and capable man who has a bad system will treat disease better than a careless and incapable man who has a good one. But was the system of blood-letting in itself utterly and always bad? Where is the man now living whose knowledge and experience are sufficient to warrant him in pronouncing positively "Yes" or "No"? There is no such man. Blood-letting was shamefully abused by ill-instructed and unconscientious men, and it fell into disrepute on that account; but was it the only system or theory which, after enjoying a high degree of fame, fell into oblivion and neglect? Who that has the least smattering of astronomical, philosophical, or theological knowledge but knows that system after system, like stage-players, have had their exits as well as their entrances: "have had their day, and ceased to be"? Was Newton the first astronomer—or the last? Did no one ever propound a complete theological system before Calvin, or since? Are not mental and moral philosophers as thick as apples in a Kentish orchard, and as various in appearance and flavour? It is astonishing and disgraceful that any considerable number of men can pass through a medical curriculum and come out, as many do, so stupidly and hopelessly ignorant of everything but the merest elements of that kind of practice which happens to be the orthodoxy of the moment. Is it the fault of their teachers, who possess nothing but that "hand to mouth" knowledge which satisfies the man whose sole object in life is "bread and butter"; or is it the incorrigible laziness and stupidity of the pupil, which prevents him, though walking through the most varied scenery, from looking at anything but the turnpike beneath his feet, and the stone walls which bound the highway on his right hand and on his left?

Before any man is entitled to pronounce on blood-letting as a system, he must have had an adequate personal experience of it. Mere extent and profundity of reading can never by any possibility give a man that positive and certain knowledge which is born of repeated experience alone. Not only so, but any person who at this distance of time shall select a typical representative of the blood-letting school like Cullen or Gregory, and utter any positive verdict on his practice in its entirety, by that very fact proclaims himself utterly destitute of original genius, though he should be the president of the College of Physicians *in perpetuo*. Think for one moment of the *rationale* of blood-letting in inflammation of the lungs. Here is a symmetrical pair of organs engorged with an abnormal quantity of blood, whilst the whole of the blood in the body remains exactly the same in quantity. Is it not reasonable to suppose that if the absolute quantity in the body be diminished the local engorgement will be lessened, and that in strict proportion to the total diminution? Where is the irrationality? Take any system of closed tubes containing fluid, and will not any engineer worth the name diminish a local engorgement by "tapping," if tapping be possible, either at the place of engorgement or elsewhere? That is the theory, unencumbered by details, and, all modern advances in physiology and pathology notwithstanding, there is nothing about it but what is perfectly reasonable and natural. So much for the system. It is based on a sound principle, the soundness of which will probably soon lead to a revival of the practice in a modified and limited degree.

The main point for practical consideration may be thus stated : Do modern doctors, who avoid blood-letting, produce better results than the doctors of former times who practised it? The answer is indubitable and decisive : On the whole they do ; though this better condition of the practice of to-day has not been brought about entirely by abstract reasoning and the substitution of one so-called "system" for another, but by developing the intelligence, the conscience, and the independent judgment of individual medical practitioners. The probability is that if, along with the present methods, blood-letting were also occasionally resorted to, the general results would be still better than those now produced. In every age each art and science has its prevailing orthodoxy, to which the bulk of its professors adhere. Sometimes that orthodoxy will be narrow, and of little saving benefit, like blood-letting, except in a very few and well-selected cases ; but so long as each man does his utmost according to the light and energy which are in him, the lamp of progress will continue to burn, and broader and more universal truths will take the place of narrow dogmas which injure as many as they help. The setting up of one system does not necessarily exclude every previous system ; but the multiplication of capable and thoroughly trained men subjects every system to a more complete and searching criticism. The wise man is he who, being well armed for the conflict, shall use that particular weapon which the circumstances of the moment demand. In the words of a famous philosopher—"Better is a bad theory than none : happy is the man who has the best."

*(To be continued.)*

